# Vestibular Lesion-Induced Developmental Plasticity in Spinal Locomotor Networks during *Xenopus laevis* Metamorphosis

**DOI:** 10.1371/journal.pone.0071013

**Published:** 2013-08-12

**Authors:** Anna Beyeler, Guillaume Rao, Laurent Ladepeche, André Jacques, John Simmers, Didier Le Ray

**Affiliations:** 1 Université de Bordeaux – CNRS UMR 5287 (INCIA), Bordeaux, France; 2 Aix-Marseille Université – CNRS UMR 7287 (ISM), Marseille, France; The Research Center of Neurobiology-Neurophysiology of Marseille, France

## Abstract

During frog metamorphosis, the vestibular sensory system remains unchanged, while spinal motor networks undergo a massive restructuring associated with the transition from the larval to adult biomechanical system. We investigated in *Xenopus laevis* the impact of a pre- (tadpole stage) or post-metamorphosis (juvenile stage) unilateral labyrinthectomy (UL) on young adult swimming performance and underlying spinal locomotor circuitry. The acute disruptive effects on locomotion were similar in both tadpoles and juvenile frogs. However, animals that had metamorphosed with a preceding UL expressed restored swimming behavior at the juvenile stage, whereas animals lesioned after metamorphosis never recovered. Whilst kinematic and electrophysiological analyses of the propulsive system showed no significant differences in either juvenile group, a 3D biomechanical simulation suggested that an asymmetry in the dynamic control of posture during swimming could account for the behavioral restoration observed in animals that had been labyrinthectomized before metamorphosis. This hypothesis was subsequently supported by *in vivo* electromyography during free swimming and *in vitro* recordings from isolated brainstem/spinal cord preparations. Specifically, animals lesioned prior to metamorphosis at the larval stage exhibited an asymmetrical propulsion/posture coupling as a post-metamorphic young adult. This developmental alteration was accompanied by an ipsilesional decrease in propriospinal coordination that is normally established in strict left-right symmetry during metamorphosis in order to synchronize dorsal trunk muscle contractions with bilateral hindlimb extensions in the swimming adult. Our data thus suggest that a disequilibrium in descending vestibulospinal information during *Xenopus* metamorphosis leads to an altered assembly of adult spinal locomotor circuitry. This in turn enables an adaptive compensation for the dynamic postural asymmetry induced by the vestibular imbalance and the restoration of functionally-effective behavior.

## Introduction

Rhythmic movements of animals arise from coordinated assemblies of local neuronal networks, so-called “central pattern generators” (CPGs), which produce rhythmically-recurring patterns of motor output [1], [2] that are continuously adjusted by sensory information [3]. Amongst such sensory cues in vertebrates, vestibular information is particularly involved in the control of locomotor and postural behavior [4] as well as corrective eye movements [5].

Vestibular lesioning results in dramatic impairments of motor functions that are progressively restored in various animal species as well as in humans. For example, an acute ablation of vestibular endorgans or vestibular nerve transection on one side (unilateral labyrinthectomy, UL) causes deficits in both postural and oculomotor reflexes (for a review, see [6]) that recover after several weeks. Vestibular compensation, which has been proposed to account for such functional restorations in terrestrial species (see [7]), consists of a gradual re-equilibration of activity in the brainstem vestibular nuclei of the two sides and implicates the use of body proprioceptive information ascending from the spinal cord (see [8]). However, although such compensatory plasticity has been extensively studied in brainstem nuclei, surprisingly very little is known about the long-term effects of a vestibular lesion on downstream locomotor networks in the spinal cord.

During metamorphosis, *Xenopus laevis* undergoes a complete restructuring of its biomechanical apparatus that is paralleled by the progressive emergence of adult patterns of neural commands for limb-based locomotion [9], [10] through a profound reorganization of the spinal circuitry responsible for both propulsive and postural functions [11]. In contrast, the vestibular sensory system, including the otic organs [12], vestibular secondary neurons and their ipsi- and contra-lateral spinal projections, is already established both anatomically and physiologically by the time of metamorphosis onset [13], [14]. This differential timetable therefore provides a unique developmental situation in which motor circuitry is undergoing *de novo* remodeling while one of its major sources of sensory input remains basically unaltered.

Here, by taking advantage of the secondary, and complete, phase of spinal development that occurs during *Xenopus* metamorphosis [11], we investigated the impact of a UL-induced imbalance in vestibular sensory information on the subsequent development and functional organization of the adult locomotor networks. To this end, both free swimming behavior and the underlying spinal motor programs were compared in two groups of young adult frogs subjected to UL: (i) juveniles previously lesioned in the pre-metamorphic tadpole (at stage 54; [15]) and whose adult spinal circuitry had therefore emerged under conditions of unbalanced vestibular influences, and (ii) juveniles with UL performed immediately after metamorphosis (at stage 66) and thus with locomotor spinal circuitry that had already been established in the presence of normal, bilaterally-symmetrical vestibular inputs. We report that, although UL induced similar acute behavioral deficits in the two lesioned groups, the former recovered near-normal locomotor performance by the time metamorphosis was completed, whereas swimming in the latter remained permanently impaired. Simulations using a 3D biomechanical model of a labyrinthectomized animal supported by a combination of *in vivo* and *in vitro* electrophysiology indicated that the locomotor recovery specific to UL54 juveniles could be attributable to asymmetric compensatory changes during metamorphic development in the spinal lumbo-thoracic circuitry responsible for propulsion/posture coupling. Our results thus highlight both the capacity for effective behavioral adaptability in metamorphosing *Xenopus* and the precision with which associated developmental plasticity occurs within the underlying spinal networks.

## Methods

Experiments were performed on the South African clawed toad *Xenopus laevis* of either sex, bred from an in-house laboratory colony raised at room temperature. All procedures complied with the National Charter on Ethics of Animal Experiments of the CNRS, and the protocol was approved by the Animal Care and Use Committee of the University of Bordeaux (Permit number 3301100012-A). All surgery was performed under tricaine methanesulfonate anesthesia, and attention was paid to minimizing the number of animals used in the experiments.

### Animal Surgery: Unilateral Labyrinthectomy (UL)

Vestibular endorgans on the right side were surgically ablated in both stage-54 tadpoles (UL54) just prior to the onset of the metamorphic period and stage-66 juvenile adults (UL66) immediately at the end of metamorphosis [15]. For this operation, animals were anesthetized with tricaine methanesulfonate (0.1% MS-222, Sigma-Aldrich) and the lesion was performed under visual control (magnification x16) in frog artificial cerebrospinal fluid (ACSF; in mM: 95 NaCl, 3 KCl, 30 NaHCO_3_, 2.5 CaCl_2_, 0.75 MgCl_2_, 0.5 NaH_2_PO_4_ and 11 C_6_H_12_O_6_, pH adjusted to 7.4) at 6°C. To access the vestibular endorgans, a 2-mm rostrocaudal incision was made in the skin over the right otic capsule and through the underlying capsule itself with fine ophthalmic scissors (Moria) under microscope control. All the vestibular endorgans (utricle, saccule, semicircular canals, and ganglion of Scarpa) of that side were carefully removed with fine forceps (see also Fig. 1A of Lambert et al., 2013), and 2 mL of ACSF were injected into the remaining capsule to remove residual tissue and calcium precipitate. After recovery from anesthesia, operated animals were placed in separate aquaria for 4 weeks to continue their development: ∼45% of lesioned stage-54 tadpoles metamorphosed successfully, while ∼75% of the lesioned stage-66 juvenile frogs survived and continued to mature into adulthood. The completeness of UL conducted on stage 66 animals and the absence of vestibular endorgan regeneration during metamorphosis in UL54 juveniles was verified during the subsequent dissection of the central nervous system for *in vitro* experimentation (see below), and only animals with an unequivocally complete lesion were kept for analysis in this study. A further group of juvenile adults which were not subjected to the vestibular lesion served as intact controls. The results reported in this study were collected from 18 intact, 16 UL54 and 15 UL66 juvenile frogs (see Table 1).

**Figure 1:**
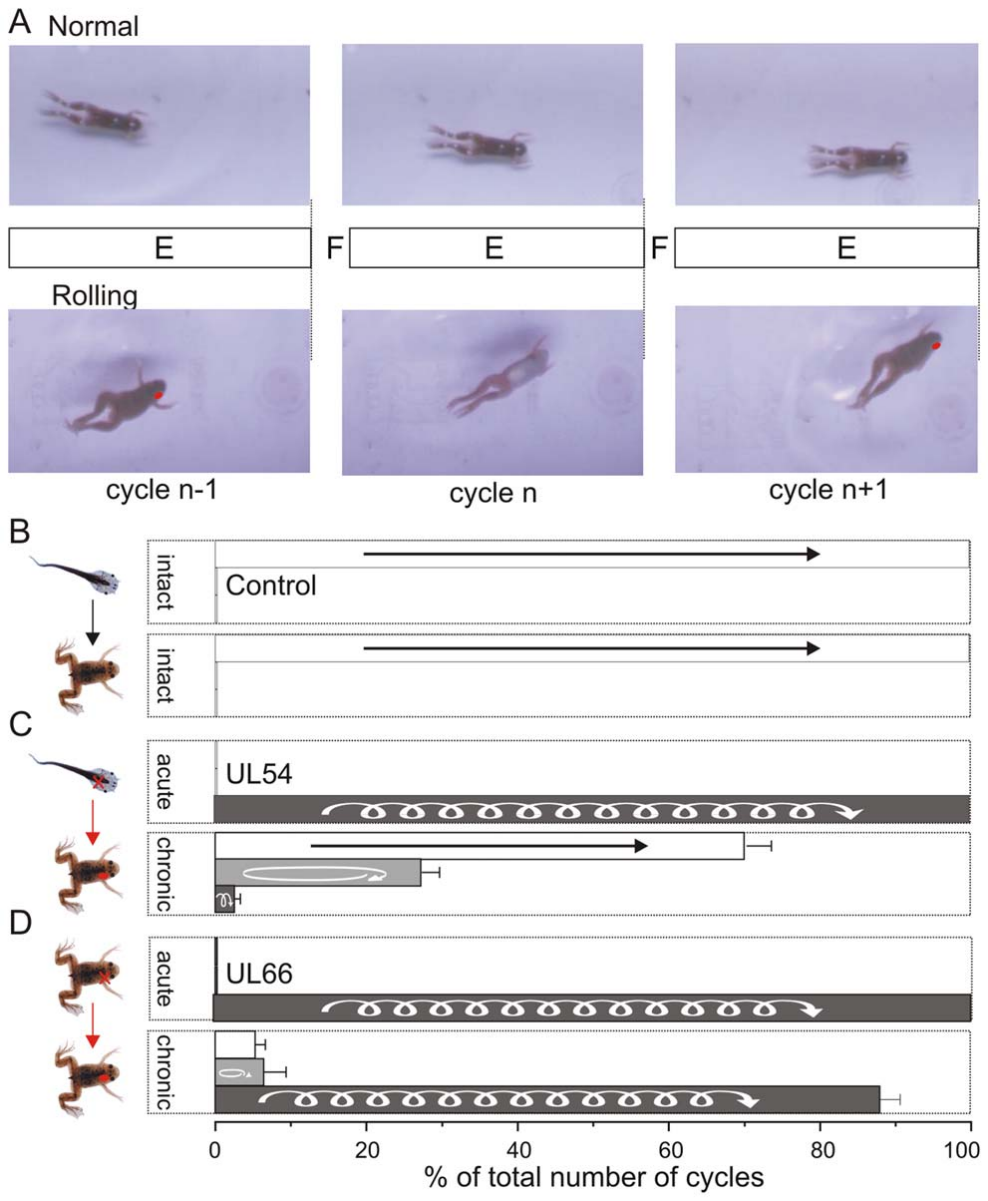
A unilateral labyrinthectomy at pre- or post-metamorphosis leads to distinct degrees of locomotor impairment in freely-behaving juvenile frogs. **A.** Images of the hindlimb extension phase (near its termination) during three consecutive cycles of normal swimming (top) and rolling behavior (bottom) expressed by control and unilateral labyrinthectomy (UL; red dot)-lesioned stage 66 *Xenopus*. Each cycle consisted of alternate limb flexions (F) and extensions (E). UL-induced rolling behavior consisted of a semi-complete rotation of the animal around its longitudinal body axis during each hindlimb extension. **B–D.** Swimming behavior of intact control animals (n = 6) before and after metamorphosis (**B**), and acute and chronic effects of a right-side UL performed at stage 54 before (n = 9, UL54, **C**) or at stage 66 after (n = 12, UL66, **D**) metamorphosis. Histograms show the percentage of swim cycles in which normal rectilinear (unfilled), circling (light grey) or rolling (dark grey) trajectories were expressed in each animal group. Error bars indicate SEM.

**Table 1:** Numbers of juvenile frogs analyzed in different experiments.

	Kin	EMG	ENG	Multi	Total
Intact	4	2	4	8	18
UL54	7	0	5	4	16
UL66	6	0	5	4	15

Distribution of experimental animals. Kin: kinematic analysis during free swimming; EMG: electromyographic recordings during free swimming; ENG: electroneurographic recordings during *in vitro* fictive swimming; Multi: combination of tests.

### Locomotion and Posture Analysis

**Figure 2:**
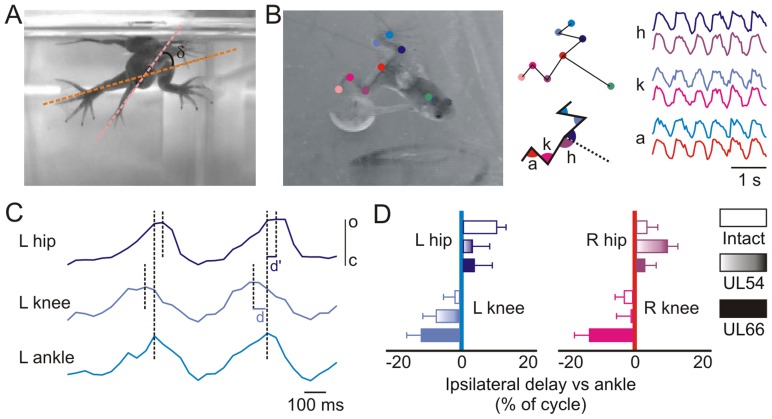
Unilateral labyrinthectomy does not affect hindlimb intersegmental coordination during swimming. **A**. Both UL54 and UL66 juvenile frogs showed a strong caudo-rostral body twisting towards the lesioned side, which was measured as the angle δ (black arc) between the eye (pink line) and knee (orange line) axes. **B.** Colored markers placed on the three main joints of both hindlimbs and on the back along the axis (upper schematic) enabled bilateral angular variations of the hip, knee and ankle to be measured (lower schematic). Each angular variation was then rectified and plotted against time (traces at extreme right) to allow delay measurements. Color code: blue, left hindlimb, purple, right hindlimb. **C**: Detail of ankle, knee and hip angular variations on the left side over two consecutive cycles. The knee-ankle delay (d) and hip-ankle delay (d’) were then calculated between maximal angular values (corresponding to the maximal joint aperture) for each cycle, and for both hindlimbs. o: open joint; c: closed joint. **D.** Knee and hip movement delays relative to maximal ankle excursion expressed as a percentage of cycle duration. No significant differences were found between control (unfilled) and UL54 (shaded) or UL66 (filled) juveniles. Error bars indicate SEM.

Animals (10 intact, 10 UL54 and 10 UL66 juveniles) were video-recorded separately for 15 min using a high speed CCD camera (Dragonfly Express) with *Fly Capture* software (Point Grey Research Inc.) at 50 frames·s^−1^, while behaving freely in a Plexiglas aquarium (23×40 cm with 5 cm depth) containing approximately 5 L of water. In these conditions, animals could swim without touching any part of the tank (e.g., Figure 1A). However, swim episode cycles were not analyzed when such contacts eventually occurred. Video images were recorded from a vertical view above the water tank and subsequently visualized and analyzed on a computer with free *Image J* software (W.S. Rasband, US National Institutes of Health, http://rsb.info.nih.gov/ij/). In a first step, an animal’s locomotor behavior was classified globally as ‘normal’, ‘circling’ or ‘rolling’ according to its manner of displacement during swimming (Figure 1). In ‘normal’ swimming, an animal maintained its dorsal side upward with a stable body orientation, whereas ‘rolling’ was characterized by continuous body rotations around the longitudinal axis toward the lesioned side, due to the animal’s inability to maintain horizontal equilibrium. In ‘circling’ behavior, the frog also turned continuously in an ipsilesional direction, but now in the horizontal plane. The number of locomotor cycles contributing to each of these three trajectory types was then counted. In a second step (Figure 2B–D), using a manual tracking plug-in supplied by F.P. Cordelières (Institut Curie, France), the *x* and *y* coordinates of the hindlimb joints were determined visually by mouse-clicking on individual video frames. Joint angles were calculated with *Excel* (Microsoft) in both static (stationary) and dynamic (spontaneous displacement; e.g., Figure 2B,C) behavioral conditions. Static posture was assessed by measuring the angles of each of the three main joints (hip, knee, ankle) of both hindlimbs, as well as the body axis angle taken from three points located respectively at the midpoint between the eyes, at mid-trunk, and at the sacrum (for example, intact animals present a back angle of ∼180°, corresponding to a near linear body axis). In addition, frontal images were used to determine the degree of body twist in static UL animals, measured as the angle between a first line passing through the two eyes and a second line adjoining the knees (for example, see angle δ in Figure 2A). Unfortunately, it was technically impossible to track this angle during actual swimming. In order to facilitate comparisons between animals, locomotor kinematic analyses were performed exclusively on episodes of straight-forward (rectilinear) swimming in normal (intact) and rolling animals, which thereby minimized the variability associated with any intentional turning behavior, as noted previously for intact juvenile frogs [11]. Episodes of circling behavior were excluded from the analysis since it was virtually impossible to distinguish between self- and lesion-induced movements. For rectilinear swimming kinematics, the delay to maximal joint opening for the two hindlimbs was measured from the rectified joint angular variations (e.g., Figure 2C for the left leg) and plotted against the time to maximal ankle angular variation, which usually occurred near mid-cycle (Figure 2C,D). Mean values calculated from the different animal groups (Table 2) were compared with one-way ANOVA and Tukey post-test using *SigmaPlot 11* software (Systat Software Inc.) and mean values were considered significantly different at *p*<0.05.

**Table 2:** Unilateral labyrinthectomy-induced alterations in static posture of juvenile *Xenopus*.

angles (°)		Back	R hip	L hip	R knee	L knee	R ankle	L ankle
Group	intact [6]	184±1	98±2	97±2	76±2	79±2	83±3	80±2
	UL54 [5]	148±2	73±20	103±4	59±9	69±12	70±6	72±17
	UL66 [6]	173±4	86±4	102±8	84±9	83±13	83±10	87±14
ANOVA: ***p*** value (power)		<0.001	<0.05	0.63 (0.05)	0.12 (0.27)	0.5 (0.05)	0.27 (0.11)	0.29 (0.01)
Tukey’s	intact vs UL54	***	*	–	–	–	–	–
post-test	intact vs UL66	ns	ns	–	–	–	–	–
	UL54 vs UL66	**	ns	–	–	–	–	–

Back and limb joint angles on the left (L) and right (R) sides were measured in stationary intact and lesioned juveniles. The number of animals in each group is indicated in parentheses. A unilateral labyrinthectomy performed before metamorphosis caused larger subsequent alterations in static posture than a post-metamorphic UL. Note that large individual variations among the UL54 group for the R hip angle (indicated by high SEM value compared to the two other groups) were responsible for the low ANOVA power (given in parentheses for non-significant ANOVA tests). The number of animals in each group is indicated in brackets. ns: non-significant;

*** p<0.001;

** p<0.01;

* p<0.05.

### Electromyographic Recordings from Freely Behaving Juvenile *Xenopus*

Electromyographic (EMG) activity of dorsal trunk and hind leg muscles was recorded using pairs of 50 µm insulated wire electrodes, implanted under light anesthesia after a small incision had been made in the overlying skin. Simultaneous EMG recordings were made bilaterally from the third myomere of the postural back muscle *dorsalis trunci* [16] and the ankle extensor *plantaris longus* [17], [18] in 6 intact, 4 UL54 and 3 UL66 animals during free swimming. The electrodes were connected through a grounded cable to a differential ‘Model 1700’ AC amplifier (AM-System Inc.) and signals were digitized at 5 kHz through a CED Micro 1401 interface (Cambridge Electronic Design) and stored on computer for later analysis. As for the kinematic analyses, only EMG signals recorded during forward rectilinear swimming were analyzed [11].

### Isolated Brainstem/Spinal Cord Preparation for Extracellular Recordings

**Figure 3:**
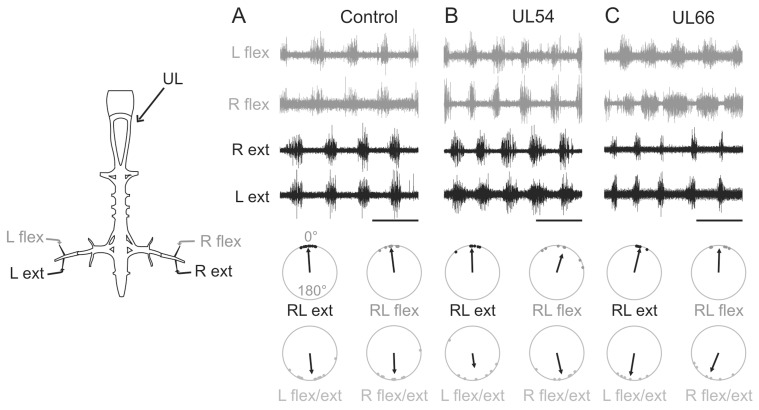
Neither pre- nor post-metamorphic UL alters hindlimb motor burst coordination *in vitro*. Sample recordings from left (L) and right (R) hindlimb extensor (ext) and flexor (flex) motor nerves during fictive rectilinear swimming in isolated brainstem/spinal cord preparations from intact (**A**), UL54 (**B**) and UL66 (**C**) juveniles. Schematic at left indicates recording electrode positions. The circular plots below each raw recording panel illustrate the corresponding phase relationships of locomotor-related activity in bilateral flexor (LR flex), extensor (LR ext) and ipsilateral left and right flexor/extensor activity (L flex/ext and R flex/ext, respectively). Measurements were pooled for each group. Individual dots represent the mean activity phase value for one animal, while the direction and length of each vector indicate respectively that population’s grand mean phase value and the concentration of individual phase values around that mean. An upward (vertical) projecting vector (0°) indicates burst synchrony, whereas a downward pointing vector (180°) indicates burst alternation. Note the preservation of a similar coordination in all three groups, consisting of bilateral synchrony between homologous extensor or flexor motor bursts, and ipsilateral alternation between antagonistic flexor/extensor bursts. Scale bars: 1s.

Dissection and electrophysiological recording procedures were similar to those described previously [11]. Briefly, at the end of metamorphosis (8 UL54 and 12 intact juveniles) or at week four thereafter (8 UL66 frogs), the dorsal cranial skin and cartilage was opened under deep anesthesia with tricaine methanesulfonate (MS-222) and the forebrain rapidly removed above the rhombencephalon. The spinal cord and brainstem were then dissected out together with the thoracic ventral roots and identified nerve branches that innervate the flexor *tibialis anterioris* and the extensor *plantaris longus* muscles of both hindlimbs (e.g., Figure 3). It is noteworthy here that the presence of the brainstem in juvenile frog *in vitro* preparations is necessary for the spontaneous production of locomotor-related output from the spinal cord [9], [11]. Presumably, the brainstem of the isolated *Xenopus* CNS is able to provide sufficient tonic signals for spinal motor network activation that in other classical vertebrate preparations usually require supplementing by exogenous pharmacological stimulation (for a review, [19]).

The isolated brainstem/spinal cord preparation was then transferred into a recording chamber and submerged in oxygenated frog ACSF at 18–20°C. Extracellular suction electrodes were used to record motor activity from the left and right thoracic ventral roots, while Vaseline-insulated wire electrodes were used to record *en passant* from the distal ends of the bilateral hindlimb extensor and flexor motor nerve branches. Ventral root and nerve signals were amplified with differential AC amplifiers Model 1700 (AM-System), then were directed at 7 kHz to a computer through a CED Micro 1401 interface for storage and later analysis using *Spike2* software (CED). All spinal motor output patterns, including those related to swimming, occurred spontaneously without additional mechanical, chemical or electrical stimulation.

### Electrophysiological Data Analysis and Statistics

All analyses of electrophysiological recordings were performed with homemade scripts running under *Spike2* software (script language, CED). Only rectilinear real or fictive swimming episodes, defined by the synchronous onsets of hindlimb extensor activity alternating with synchronous flexor muscle or motor nerve bursts [11], were analyzed. Motor burst onsets were detected on the raw traces, and data were then transferred to *Oriana* software (Kovach Computing Services) for circular phase analysis of the temporal relationship between activities in selected pairs of muscles (for *in vivo* recordings) or ventral roots and limb nerves (*in vitro* recordings). This analysis gave the mean vector *µ* in degrees (°) and its length *r.* Two uniformity tests were used to examine the distribution of phase values: the Rayleigh’s test (Z), which tested the null hypothesis that the data were uniform, i.e., randomly distributed throughout 360°, and the V-test (*u*), which tested the null hypothesis of uniformity against the alternative of a non-uniform distribution with a specified mean direction (0° or 180° corresponding to strict synchrony or alternation, respectively, and denoted by *u*(0°) or *u*(180°) in the data analysis). Phase values were plotted as the grand mean of the individual means of relative burst onsets per animal. Hindlimb locomotor burst periods and durations were also measured during ‘rectilinear’ fictive swimming in all three groups of animals and compared with one-way ANOVA and Tukey’s post-test. The results of all statistical tests are collated in Tables 3, 4, 5.

**Table 3:** Circular statistical analysis of the phase relations between lumbar and thoracic ventral root motor bursts *in vitro*.

	Control (9)	UL54 (8)	UL66 (8)
L-R Flex	Z = 5.80;p<0.001 *u*(0)° = 4.34; p<0.001	Z = 4.34;p<0.01 *u*(0)° = 2.95; p<0.001	Z = 4.38;p<0.01 *u*(0)° = 2.96; p<0.001
L-R Ext	Z = 8.56;p<0.001 *u*(0)° = 4.13; p<0.001	Z = 6.39;p<0.001 *u*(0)° = 3.57; p<0.001	Z = 5.91;p<0.001 *u*(0)° = 3.37; p<0.001
L Flex-Ext	Z = 5.56;p<0.001 *u*(180)° = 3.30; p<0.001	Z = 3.18;p<0.05 *u*(180)° = 2.43; p<0.01	Z = 4.16;p<0.01 *u*(180)° = 2.85; p<0.001
R Flex-Ext	Z = 5.07;p<0.01 *u*(180)° = 3.10; p<0.001	Z = 3.20;p<0.05 *u*(180)° = 2.50; p<0.01	Z = 4.14;p<0.01 *u*(180)° = 2.65; p<0.01
L-R Th2	Z = 4.38; p<0.01* u*(0)° = 2.87; p<0.001	Z = 4.52; p<0.01* u*(0)° = 3.01; p<0.001	Z = 3.74; p<0.05* u*(0)° = 2.68; p<0.001
R Th2-Ext	Z = 5.83;p<0.001 *u*(0)° = 2.42; p<0.001	***Z = 1.34; p = 0.29 u*** **(0)° = 1.29; p = 0.11**	Z = 3.80;p<0.01 *u*(0)° = 2.58; p<0.01
L Th2-Ext	Z = 6.96; p<0.001* u*(0)° = 3.59; p<0.001	Z = 4.27; p<0.01* u*(0)° = 2.55; p<0.01	Z = 3.64; p<0.05* u*(0)° = 2.67; p<0.01

Coordination patterns between left (L) and right (R)-side flexor and extensor fictive locomotor activities, and with bilateral thoracic (Th2) motor bursts. The Rayleigh (Z) test was used to verify non-uniformity of the circular distributions, and the V-test was used to compare distributions with a selected direction (either 0°, *u*(0°), for synchrony or 180°, *u*(180°), for alternation). Bold characters highlight statistically random distribution.

**Table 4:** Cycle periods and burst durations in hindlimb kicking motor patterns *in vitro*.

Cycle period (s)		L ext	R ext	ANOVA and Tuckey’s post-test on L-R ext (power)		L flex	R flex	ANOVA and Tuckey’s post-test on L-R flex (power)	
Group	intact [9]	0.52±0.04	0.53±0.03	ns (1)		0.54±0.04	0.53±0.03	ns (1)	
	UL54 [8]	0.52±0.02	0.53±0.02	ns (1)		0.55±0.02	0.53±0.03	ns (1)	
	UL66 [8]	0.60±0.03	0.62±0.04	ns (1)		0.60±0.03	0.66±0.04	ns (1)	
ANOVA: ***p*** value (power)		0.20 (0.15)	0.19 (0.16)			0.19 (0.16)	0.07 (0.33)		
Tukey’s–	intact vs UL54	–	–			–	–		
post-test	intact vs UL66	–	–			–	–		
	UL54 vs UL66	–	–			–	–		
**Burst duration (s)**		**L ext**	**R ext**	**ANOVA and Tuckey’s post-test (L-R ext)**		**L flex**	**R flex**	**ANOVA and Tuckey’s post-test (L-R flex)**	
Group	intact [9]	0.13±0.01	0.16±0.01	ns (1)		0.25±0.01	0.24±0.02	ns (1)	
	UL54 [8]	0.15±0.01	0.16±0.01	ns (1)		0.29±0.01	0.25±0.01	ns (1)	
	UL66 [8]	0.22±0.01	0.28±0.02	ns (1)		0.28±0.02	0.33±0.02	ns (1)	
ANOVA: ***p*** value (power)		<0.001	<0.001			0.30 (0.84)	<0.01		
Tukey’s–	intact vs UL54	ns	ns			–	ns		
post-test	intact vs UL66	***	***			–	*		
	UL54 vs UL66	***	***			–	ns		

UL performed either pre- or post-metamorphosis caused no significant changes in the fictive locomotor cycle period, whereas the durations of extensor bursts were significantly altered following a post-metamorphic UL. Note also a slight increase in ipsilesional flexor burst durations in UL66 juveniles compared to both intact and UL54 animals. The number of animals in each group is indicated in brackets and the power of non-significant ANOVA tests in parentheses.

**Table 5:** Circular statistical analysis of the phase relations between hindlimb extensor and dorsal muscle activities *in vivo*.

	Control (9)	UL54 (8)	UL66 (8)
L-R *dt*	Z = 21.32; p<0.001* u*(0)° = 6.50; p<0.001	Z = 4.05; p<0.05** ***u*** **(0)° = 1.25; p = 0.11**	Z = 12.58; p<0.001* u*(0)° = 5.01; p<0.001
L-R *pl*	Z = 23.96; p<0.001* u*(0)° = 6.92; p<0.001	Z = 19.01; p<0.001* u*(0)° = 6.10; p<0.001	Z = 12.62; p<0.001* u*(0)° = 5.01; p<0.001
L *dt*-*pl*	Z = 22.68; p<0.001* u*(0)° = 6.73; p<0.001	Z = 15.98; p<0.001* u*(0)° = 5.48; p<0.001	Z = 12.46; p<0.001* u*(0)° = 4.93; p<0.001
R *dt*-*pl*	Z = 18.34; p<0.001* u*(0)° = 6.06; p<0.001	**Z = 2.61; p = 0.07** *** u*** **(0)° = 1.35; p = 0.09**	Z = 12.50; p<0.001* u*(0)° = 4.97; p<0.001

Coordination patterns between left (L)- and right (R)-side *plantaris longus* (*pl*) and *dorsalis trunci* (*dt*) muscle activity recorded by EMG. Non-uniformity of circular distributions was verified by the Rayleigh (Z) test, and the V-test (*u*(0°)) was used to assess burst synchrony. Bold characters highlight statistically random distributions.

### Biomechanical Simulations of Lesioned Animals and Swimming Behavior

**Figure 4:**
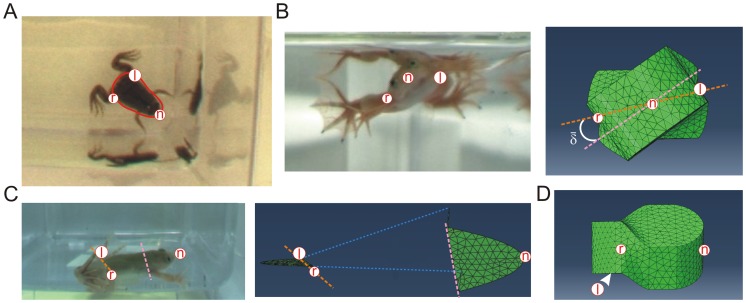
3D model of a UL juvenile with twisted body trunk. Geometrical model of a lesioned animal’s trunk using the 3D finite element (FE) method, based on anatomical characteristics and the assumption that the main body/water interactive forces occur at the level of the trunk (**A**). Once the initial FE model body was built, a 37° torsion (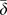) was applied in the antero-posterior axis (**B** right) in order to simulate the mean body twist towards the lesioned side observed in UL juveniles (**B** left). Red markers n (nose), r (right hip) and l (left hip) indicate model orientation and together with the two dashed lines (see **C**), illustrate the model’s torsion. **C:** The two artificial front and rear rigid body components, respectively simulating the scapula and pelvis belts, were linked by an elastic portion to which the torsion was applied. The pink and orange dashed lines indicate the medial plans of the front and rear rigid body components (green plans), respectively, while the l and r red markers correspond to the linear left and right limits of the rear medial plan, and the n marker indicates the front of the anterior plan. *Dorsalis* muscles were simulated by two actuators (blue dashed lines) placed on both sides of the antero-posterior axis between the two rigid components. **D:** Lateral (right) view of the twisted FE model corresponding to UL-induced distortion in juvenile frogs. Arrowhead indicates that the left hip marker (l) is on the non-visible side of the model.

**Figure 5:**
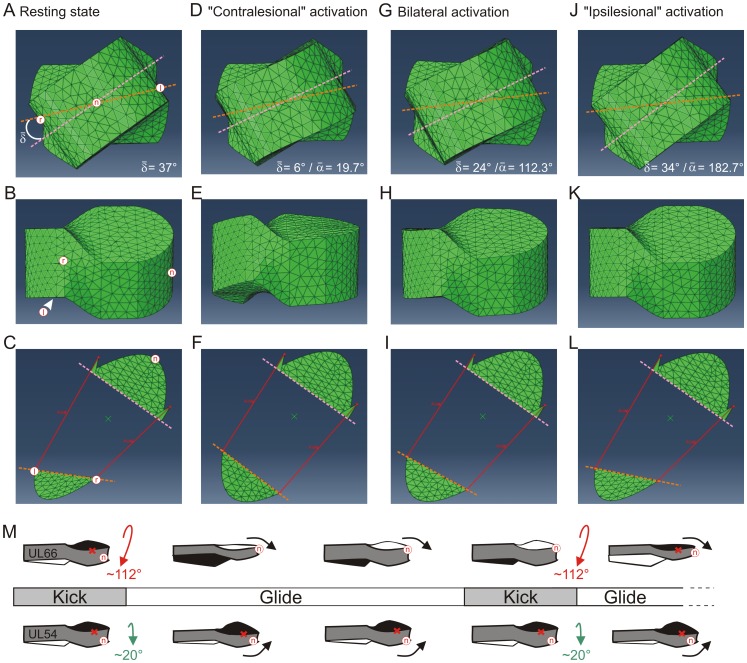
Different patterns of actuator activation induce different body distortions and resultant changes in aquatic displacement. **A–L:** Frontal (A, D, G, J), lateral right (B, E, H, K) and exploded view (C, F, I, L) of the FE model at initial resting state (A–C) and at the end of either a left side (“contralesional”; D–F), symmetrical (Bilateral; G–I) or right side (“ipsilesional”; J–L) activation of the longitudinal actuators. The 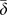 angle at the end of each simulation and the resulting angular displacement during propulsion (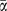; obtained from the subsequent dynamic simulation) are indicated in panels D, G and J. Arrowhead in **B** indicates that the left hip marker (l) is on the non-visible side of the model. **M**: Theoretical explanation of lesioned animal movement according to simulation results. A high angular velocity associated with the strong body torsion of animals with symmetrically-activated postural back muscles (top) induces a complete disequilibrium of the body (red arrow; 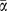 value taken from panel **G**) during each hindlimb extension, as observed experimentally in UL66 juvenile frogs. In contrast and in correspondence with UL54 juvenile behavior, a lower angular velocity associated with the much reduced body torsion of animals with an asymmetrical propulsion/posture coupling (bottom) causes only a slight disequilibrium (green arrow; 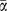 taken from panel **D**) that is subsequently compensated for by passive water reaction forces (black arrows) during the remainder of the kick cycle. Color code: black, dorsal, white, ventral, and grey, lateral sides of the body.

Two simulations using a simple model of a UL juvenile frog were developed to evaluate the influence of postural back muscle activation on the animal’s posture and during swimming behavior (Figures 4 and 5). The first simulation used a 3D finite element (FE) model (see [20] for another application of the FE method in frog swimming) to investigate the influence of a symmetrical versus asymmetrical back muscle activation on the general body shape of a juvenile frog displaying the trunk twisting typically associated with UL-induced skeletal deformation [21]. The values of the final longitudinal body torsion obtained from this first simulation were then implemented into a second simulation, based on rigid body equations of motion, in order to test the effects of body shape on swimming kinematics.

Using Simulia Abaqus 6.10 software (Dassault), a simplified representation of a posturally-twisted UL juvenile frog was created as a geometrical shape comprising 14800 tetraedric elements (based on average *Xenopus* trunk dimensions at <2 months post-metamorphosis: length = 15 mm, width = 10 mm and height = 7 mm; see Figure 4). The 3D model (Figure 4B–D) consisted of two rigid components, representing the animal’s relatively non-deformable shoulder and hip bony regions, embedded within the simplified representation of the twisted body format resulting from UL. The actual angle formed by 2 lines transecting the eyes and in turn the two knees was measured to estimate the mean twisting deformation along the body axis caused by UL in both UL54 and UL66 juvenile groups. Based on these physiological measures (e.g., Figure 2A), a 37° longitudinal torsion was applied between the model’s front and rear components in such a way that the global shape corresponded to the average postural bending towards the lesioned side observed in UL animals (Figure 4B). The internal interconnection between the two rigid components was modeled as isotropic linear elastic material, the stiffness of which was arbitrarily set to 0.04 GPa (corresponding to a flexible rubber with a compliance that is classically used to approximate that of animal tissue). Postural back muscles were incorporated into the model as two linear force-producing actuators traversing the length of the animal. As a realistic anatomical representation [16], these actuators were inserted on each side of the midline and attached to the anterior and posterior rigid components (see Figure 4C). Consistent with *in vivo* electromyographic swimming data, the force profile consisted of a 0.25 s linear ramp from 0 to 10 N followed by a further 0.25 s at a constant 10 N. Different patterns of postural muscle activation were simulated by differently-applied forces to the two linear actuators, and the effects of a symmetrical versus non-symmetrical activation were compared (Figure 5). In the asymmetrical configuration only one actuator was active, either on the ‘contralesional’ or ‘ipsilesional’ side (*i.e.*, with respect to the side of bending), whereas in the symmetrical configuration, both actuators were simultaneously and equally activated. The resulting deformation in each case was analyzed at the end of the FE simulation by measuring the longitudinal torsion between the front and rear regions of the body model (taken as the angle 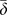 between front and rear rigid components; see dashed lines in Figure 4C).

This information was subsequently used in a dynamic simulation to estimate the changes in the animal’s body orientation in the frontal plane during swimming. Based on the approximation that only the hydrodynamic lift force plays a significant role in this plane, regular equations of motion were solved using the ODE45 function in Matlab (Mathworks Inc.), with a 0.01 s time-step, to estimate the animal’s overall displacement in the water. Equation 1 indicates that body orientation is directly related to the animal’s inertia and the hydrodynamic lift forces:
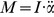
(1)where *M* is the torque generated by the hydrodynamic lift force, *I* the animal’s moment of inertia around the antero-posterior axis, and 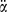 the angular acceleration of the body around this axis. The value of *M*, the moment due to the hydrodynamic lift force, was calculated with Equation 2:
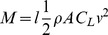
(2)where *l* represents the moment arm of the hydrodynamic force, *ρ* the fluid density, *A* the planform area, *C_L_* the lift coefficient, and *v* the animal’s speed. Because the dynamic simulation of swimming was only a validation of concept, certain assumptions were made: (i) the moment arm *l* of the hydrodynamic force was kept constant at 3.4 mm (i.e., 2/3^rd^ of the animal half width); (ii) the moment of inertia *I* was set at 5.10^−8^ kg.m^2^, corresponding to the animal representing an ellipsoidal cylinder with known dimensions and mass; and (iii) due to its global shape, with a nearly flat ventral surface and a convex dorsal surface, the juvenile body corresponds to a plane wing (NACAA 12 airfoil-type) and, therefore, aerodynamic formulae were used to estimate the remaining parameters: the amount of deformation at the end of FE simulation was used to determine both the planform area A and the angle of incidence of the model through water, which in turn was used to estimate the lift coefficient CL [22]. Based on actual recordings from freely-behaving juvenile frogs, the swim velocity was maintained at 15 cm/s throughout the simulation. The solution to Equation 1 provided a time series of the FE model angular acceleration around the antero-posterior axis during swimming. Values for the angular velocity (in °.s^−1^) and the angle formed with the horizontal axis (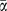) were obtained by simple mathematical integrations, The latter parameter in turn provided direct information about the mode of displacement (i.e., rolling or not) in water (note that an angular velocity of 0°.s^−1^ corresponds to swimming where the animal maintains constant upward orientation of its back throughout displacement).

## Results

### Pre- and Post-metamorphic UL have Distinct Long-term Effects on Juvenile Frog Posture and Locomotion

The spontaneous swimming and postural behavior of UL54 and UL66 animal groups were video-recorded immediately following a right-side vestibular endorgan ablation, then in each case four weeks later, in correspondence with the average duration of metamorphosis [15]. As already described in *Xenopus* [23] and other vertebrate species [7], [24], an acute UL immediately induced a characteristic impairment of locomotor and postural behavior that was never observed in normal animals at either developmental stage (Figure 1A top and B). This locomotor deficit, which was manifested as so-called “rolling” behavior (Figure 1A bottom), was similarly expressed after lesioning in both stage 54 tadpoles and stage 66 juvenile frogs (Figure 1C–D, ‘acute’ panels).

However, four weeks post-UL, striking differences were apparent in the locomotor performance of UL54 and UL66 animals (Figure 1C–D, ‘chronic’ panels). After reaching the juvenile adult stage at the end of metamorphosis, UL54 animals with their newly developed limbs expressed a largely restored locomotor capability: again, like intact control juveniles, they could maintain body equilibrium and remain dorsal-side upwards during swimming, with >70% of individual kick cycles contributing to a normal forward rectilinear trajectory (Figure 1C, ‘chronic’ panel). Although a significant proportion (27.2±9.2%) of apparently symmetrical, bilaterally-synchronous hindlimb extensions resulted in so-called “circling” behavior, UL54 animals rarely (2.6±1.8% of all kick cycles) expressed rolling behavior, and only when in a highly elevated state of arousal.

In contrast, the locomotor performance of animals in the UL66 group remained impaired 4 weeks after lesioning (Figure 1D, ‘chronic’ panel). These frogs very rarely expressed normal rectilinear swimming in the horizontal plane, and the large majority of propulsive hindlimb extensions continued to produce rolling (>85%) or horizontal circling behavior (∼10%; Figure 1D; dark and light shaded bars, respectively). Similar proportions of defective locomotor behavior in UL66 animals were observable up to 18 months after vestibular lesioning (data not shown). This lack of behavioral restoration in UL66 as compared to UL54 frogs therefore indicated that the latter’s’ locomotor network had been subjected to adaptive processes that were specific to the period of metamorphic development.

An analysis of bilateral hindlimb joint and body angles during static posture failed to explain the striking behavioral differences between the UL54 and UL66 juvenile groups at 4 weeks post-lesion. In both cases, animals showed a marked body distortion along their vertebral axis, with the anterior half of the trunk being twisted to an angle of 36.6±3.5° towards the lesioned side (e.g., Figure 2A). There was no observable difference in this torsional deformation between the UL54 and UL66 groups. However, solely UL54 juveniles exhibited a marked scoliotic vertebral curvature essentially in the horizontal plane towards the lesioned side (p<0.001; Table 2). This type of UL-induced deformation has been recently shown to derive from a persistent bilateral asymmetry in the activity of brainstem descending pathways to the spinal cord during metamorphosis [21]. In the present study, the occurrence of scoliotic skeletal alterations in UL54 but not UL66 animals was verified during subsequent dissection (see below), as was the occurrence of longitudinal vertebral twisting in both lesioned groups.

In addition, no significant bilateral variations were observed at rest in most hindlimb joint positions of UL54 compared to both intact and UL66 animals (Table 2), although a reduction (p<0.05) was found in hip angle on the ipsilesional side. Although a tendency for reduced ipsilesional knee and bilateral ankle angles was also observed in UL54 animals, the large variability within this group did not allow any significant differences to be detected (note the low power values of the ANOVA tests; Table 2). In direct contrast, however, although the swimming performance of UL66 juvenile frogs remained strongly impaired (see Figure 1D), no postural differences were found between these long-term lesioned animals and intact controls (Table 2).

During free forward swimming in intact frogs, propulsion is generated by repetitive cycles of bilaterally-synchronous hindlimb extensions (Figure 1A upper; see also [11]), with each cycle comprising coordinated sequences of knee, ankle and hip displacement on the two sides. Analyses of the knee-ankle-hip joint movement sequences in the three animal groups showed that both UL54 and UL66 animals behaved very similarly to intact frogs (Figure 2D), with no ipsi- or bilateral differences observable between groups. Thus, the kinematics of actual hindlimb movements provided no correlative explanation for the restoration of normal swimming in juvenile frogs subjected to pre-metamorphic UL, as compared to the permanent locomotor impairment in animals that had carried UL for a similar period, but subsequent to metamorphosis. Rather, these findings suggested that the locomotor propulsive mechanism itself was not implicated in the compensatory process that enabled UL54 swimming recovery.

### Neither a Pre- Nor Post-metamorphic UL Produced Significant Modification in Spinal Motor Output for Propulsion

In order to further test the hypothesis that none of the unilateral lesions significantly affected actual propulsive function, we recorded bilateral hindlimb flexor and extensor motor nerves in isolated brainstem/spinal cord preparations (Figure 3), which in the case of juvenile *Xenopus* are able spontaneously to generate the rhythmic motor patterns that normally drive swimming behavior *in vivo* [9], [11]. As is evident in the circular phase analysis of Fig. 3, such *in vitro* locomotor-related activity, which in control preparations (Fig. 3A) is known to be associated with rectilinear forward displacement in the intact animal [11], was not significantly different from the fictive limb-kick patterns expressed by the isolated brainstem/spinal cords of either UL54 (Fig. 3B) or UL66 (Fig. 3C) animals. In all three groups, rhythmic bursting in homologous left-right flexor and extensor motor nerves occurred in strict synchrony (Table 3: *u*(0°) significant for all three groups; see Methods), with ipsilateral flexor and extensor motor bursts occurring in antiphase (Table 3: *u*(180°) significant for all three groups).

These *in vitro* results therefore corresponded closely to our above *in vivo* observation that the synchronous propulsive kicking movements of the hindlimbs were similarly expressed in the three groups of juvenile frogs. Nevertheless, although the coordination of fictive limb kicking and actual movement was similar in the three groups, comparisons of cycle periods and burst durations indicated that a post-metamorphic UL affected other temporal aspects of the lumbar locomotor program produced *in vitro*. While cycle periods were not significantly different between groups, an increase in the durations of bilateral extensor bursts (p<0.001) and ipsilesional flexor bursts (p<0.01) was evident in isolated UL66 compared to intact and UL54 preparations (Table 4). In the former, however, since fictive rhythmic extensions and flexions, like the actual joint angle excursions *in vivo,* were still occurring in close bilateral synchrony and with symmetrical left-right durations, the observed differences did not comply with the persistence of rolling behavior in UL66 animals. Together, therefore, these findings further support the conclusion that the behavioral adaptation observed in post-metamorphic UL54 animals did not arise from significant modifications to the neural system responsible for generating propulsive limb movement.

### Relationship between Dynamic Propulsion/Posture Coupling and Locomotor Recovery in UL54 Juvenile Frogs: Evidence from Biomechanical Simulations

In addition to producing momentum, effective locomotor behavior depends on postural stability that requires dynamic adjustment during body displacement [25]. Thus, any inappropriate coordination between the postural and propulsive motor systems might result in incorrect locomotor movements. On this basis, therefore, we asked whether such an abnormal propulsion/posture coupling could be responsible for the permanent locomotor impairment found in UL66 juvenile frogs. Conversely, a specific compensatory adaptation of this functional interaction during metamorphosis of UL54 animals could have enabled their swimming recovery as young adults. We therefore sought to establish that an altered propulsion/posture coupling can indeed impact directly on juvenile *Xenopus* swimming behavior, and especially, whether appropriate modifications of dynamic postural control during swimming in animals with a UL-induced twist distortion could improve their locomotor performance. However, since it is impossible to access and manipulate specifically the central networks involved in the dynamic control of posture in a living animal, we turned to simplified simulations of frog swimming using a 3D model constructed from actual morphological data (see Methods for description and Figure 4).

In a first step, a finite element (FE) model of a UL-twisted juvenile frog was developed as shown in Figure 4, and initial tests were conducted to validate the effect of such a body distortion on swimming in the absence of any postural system involvement (data not shown). Then, we tested whether different patterns of activation of the two linear actuators (which simulated the bilateral longitudinal dorsal muscles of the trunk, see Methods) could have variable effects on FE model torsion and consequently lead to distinct kinematics during swimming. Because UL in *Xenopus* generates a permanent disequilibrium between left and right descending brainstem commands to the spinal cord [21], we compared the influence of a bilaterally-symmetric versus non-symmetric activation of the postural system on static body shape (Figure 5A–L). The simulations showed that a symmetrical activation of the ‘postural’ actuators caused a minor decrease of the torsion angle from 37° to 24° (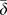; Figure 5G–I), which corresponded to a ∼35% reduction in the initial body bending (Figure 5A–C). Strikingly, however, a unilateral (asymmetric) activation applied solely to the actuator on the bent side of the model (“ipsilesional” configuration) generated a negligible torsion change from 37° to 34° (Figure 5J–L). In direct contrast, an activation of the longitudinal postural system on the opposite (“contralesional”) side only; (Figure 5D–F) induced a significantly greater change (>84%) in the shape of the virtual animal, leading to a final body torsion of <6°. These simulations thus suggested that the degree of distortion of the lesioned animal’s body might be significantly reduced when postural muscles solely on the ipsilesional side were activated concomitantly with each propulsive hindlimb extension, thereby resulting in a substantial ameliorating influence on the kinematics of the animal’s swimming.

To further test this idea, we incorporated the values of body torsion obtained from the above three FE model configurations into a dynamic simulation of real animal movement. The results demonstrated that the angular velocity generated around the antero-posterior axis by the symmetrical model was 572°.s^−1^, whereas the velocities generated by the non-symmetrical models were 913°.s^−1^ and 99°.s^−1^ for the “ipsilesional” and “contralesional” configurations, respectively. *In vivo* EMG recordings had previously shown that the *dorsalis* muscles were active for slightly less than 0.2 s during each locomotor cycle, corresponding to ∼33% of the total cycle duration [11]. From our dynamic simulation, either a symmetrical activation or an asymmetric ipsilesional activation during the 0.2 s of each hindlimb extension generate a dramatic balance deficit: calculated 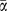 values were respectively 112.3° and 182.7° and thus were largely higher than the 90° body tilt towards the lesioned side required to induce full body disequilibrium. Consequently, this would result in a 180° rotation of the animal around its antero-posterior body axis due to body-water interactions (Figure 5M top), which in turn corresponds to the rolling behavior expressed by UL66 *Xenopus* (see Figure 1A bottom and 1D). In contrast, an asymmetric activation of the contralesional *dorsalis* muscle for 0.2 s only produced <20° tilting of the model frame during swimming (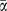 = 19.7°). This deviation was therefore considerably lower than the complete disequilibrium that is required to make the animal roll (Figure 5M bottom). Here again, these latter simulation findings were in close correspondence with our behavioral observations on UL54 animals (Figure 1C).

### UL54 Frogs Express Asymmetric Coordination between Hindlimb and Dorsal Muscle Activity During Free Swimming

**Figure 6:**
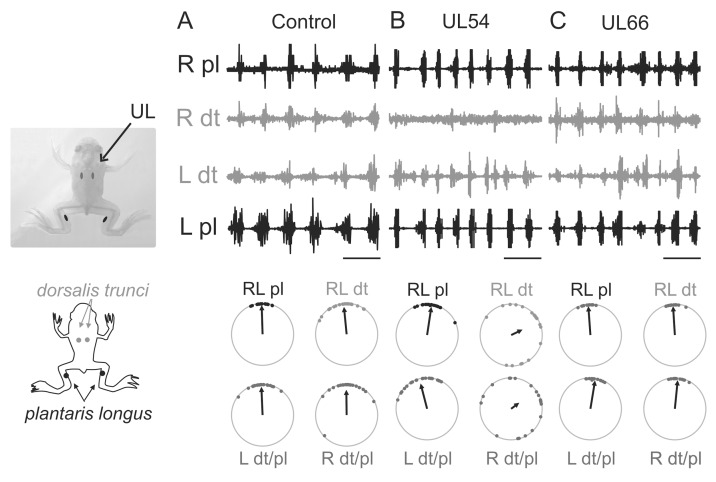
Solely a pre-metamorphic UL alters dorsal muscle/limb extensor muscle coordination in the post-metamorphic frog. Sample left (L) and right (R) electromyographic (EMG) recordings from dorsal back muscle *dorsalis trunci* (dt) and ankle extensor muscle *plantaris longus* (pl) in intact (**A**), UL54 (**B**) and UL66 (**C**) juveniles. The sites of the vestibular lesion (UL) and EMG electrode placements are shown at left. The corresponding circular plots (layout equivalent to Figure 3 except that each dot represents the mean for a single forward rectilinear swim episode) indicate the lack of bilateral *dorsalis* and right side (ipsilesional) *dorsalis*/*plantaris* coordination in the UL54 group only. Scale bars: 1s.

Our simulation data thus suggested that the expression of permanently altered swimming in UL66 animals in contrast to the restoration of close-to-normal locomotor behavior in UL54 animals could have arisen from differences in the dynamic coordination between dorsal trunk muscle contractions involved in postural control and propulsive hindlimb extensions during swimming. In order to test this theoretical prediction in the animal, bilateral EMG recordings were made from a back muscle (*dorsalis trunci*) component of the longitudinal posture system and an ankle extensor muscle (*plantaris longus*) involved in cyclic limb extensions in normal intact and UL juvenile frogs during free swimming. We previously reported [11] that after metamorphosis, the dorsal trunk muscles of intact *Xenopus* are driven directly by the lumbar CPG for hindlimb kicking during rectilinear swimming, resulting in limb extensor and back muscle contractions occurring in strict bilateral and caudo-rostral synchrony in each cycle (see also Figure 6A). Here, we found that a UL performed after metamorphosis had no effect on this pattern of muscle coordination. As evident in the raw recordings and circular analyses of Figure 6C, the phase relationships between limb extensor and back muscle EMG activity in UL66 juvenile frogs were similar to those of intact animals (cf. Figure 6A), with all mean vectors indicating phase synchrony (see also Table 5). In contrast, in UL54 juveniles a cyclic co-activation of *dorsalis trunci* and the ankle extensor muscles was virtually absent on the right, ipsilesional side of these animals during swimming (Figure 6B). As indicated by the corresponding circular plots, the phase values for right *plantaris vs dorsalis* muscle activity were randomly distributed (see also Table 5), resulting in a mean vector length that was below the random threshold (*r* = 0.35). Correspondingly, the coupling between bilateral *dorsalis trunci* was also significantly diminished (µ = 64°, *r* = 0.44), whereas the coordination between left and right *plantaris* and between contralesional (left side) *plantaris* and *dorsalis* was unaffected, with the two muscles remaining synchronously active per cycle (µ = 8°, *r* = 0.95 and µ = 346°, *r* = 0.87, respectively; Table 5). These *in vivo* EMG data therefore revealed that the characteristic bilaterally-symmetrical, in-phase coupling between propulsive hindlimb and postural dorsal trunk muscle activity found in unlesioned control animals, also persisted in UL66 frogs. In contrast, in UL54 animals this coupling pattern had become altered to an asymmetric relationship in which ipsilesional dorsal muscle activation with each propulsive limb extension had disappeared. Therefore, in correspondence with the persistent rolling versus restored straight-line swimming of UL66 and UL54 animals, respectively, these biological EMG data are in close agreement with the prognosis of our dynamic simulations (see above).

### Pre-metamorphosis UL-induced Alterations in Lumbo-thoracic Coordination are Intrinsic to the Spinal Cord

**Figure 7:**
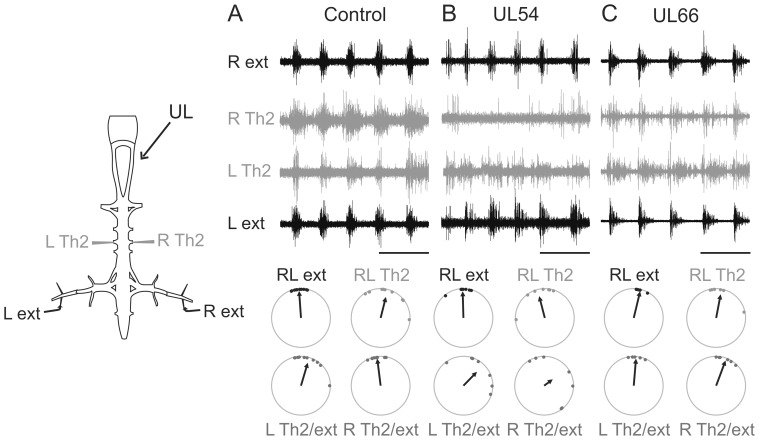
Thoraco-lumbar coordination *in vitro* is also exclusively affected by a pre-metamorphic UL. Sample simultaneous recordings from left (L) and right (R) thoracic ventral roots (Th2) and lumbar nerve branches to the left and right ankle extensor muscles (ext) during fictive rectilinear swimming in isolated brainstem/spinal cord preparations from intact (**A**), UL54 (**B**) and UL66 (**C**) juveniles. Schematic at left indicates electrode placements for nerve recordings. The corresponding circular plots (same layout as Figure 3) show that the lumbo-thoracic coordination on the right (ipsilesional) side was altered solely in isolated preparations from UL54 animals. Scale bars: 1s.

The *de novo* establishment of lumbo-thoracic propriospinal interactions during metamorphosis has been found to underlie the tight coupling of thoracic *dorsalis* motor output with each lumbar CPG-driven hindlimb extension during young adult swimming [11]. We therefore investigated in isolated brainstem/spinal cords of labyrinthectomized juveniles whether the loss of ipsilesional *plantaris*-*dorsalis* coordination in post-metamorphic UL54 animals resulted from an abnormal network construction involving this propriospinal pathway. Propulsion-related activity was recorded from hindlimb flexor and extensor nerves on both sides (as in Figure 3) and postural activity from the bilateral thoracic ventral roots that innervate the *dorsalis* muscles [11]. During episodes of spontaneous fictive rectilinear swimming, the thoracic motor nerves expressed bursting activity that was phase-coupled with ipsilateral extensor bursts in both intact and UL66 juvenile frogs (Figure 7A,C; Table 3). In contrast, in UL54 preparations this functional coupling was absent on the right (ipsilesional) side of the spinal cord (Figure 7B; Table 3). Here, thoracic nerve discharge when present was dispersed throughout the ongoing locomotor cycle (Table 3), as indicated by the mean vector length that was below the random threshold (*r* = 0.41). These *in vitro* data were therefore consistent with our *in vivo* observations suggesting that the changes in dorsal/hindlimb muscle coordination during swimming were specific to UL54 animals. Together with our previous finding of the crucial role played by lumbo-thoracic propriospinal interactions in propulsion/posture coordination in the swimming *Xenopus* frog [11], these *in vitro* results further suggested that in animals that had carried a vestibular sensory imbalance through metamorphic development, an adaptive functional reorganization occurred directly within the spinal locomotor networks themselves.

## Discussion

**Figure 8:**
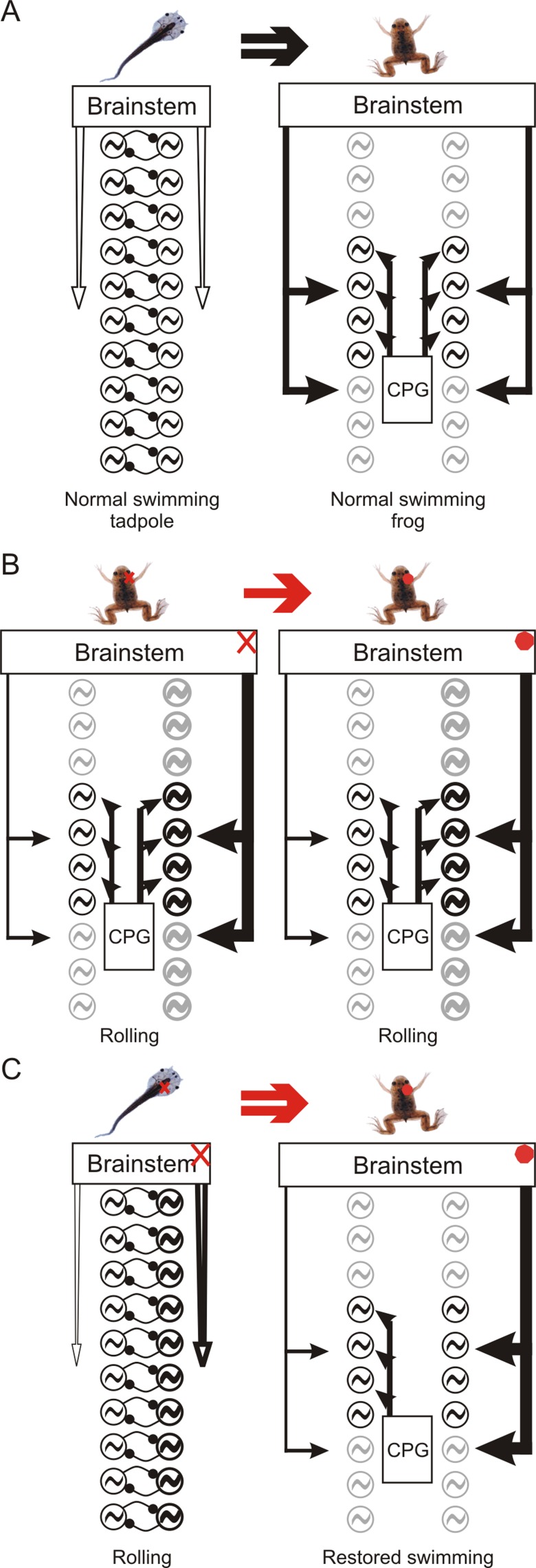
Summary of changes occurring in spinal locomotor-related networks during metamorphosis and after a right-side labyrinthectomy. **A:** Normal metamorphic modifications to spinal motor networks responsible for propulsion and dynamic postural adjustments during swimming (see Beyeler et al., 2008). Note the symmetrical left-right organization in the post-metamorphic juvenile frog. **B:** In already metamorphosed animals, UL causes asymmetry in the activity of descending brainstem commands to the spinal motor networks, producing an over-excitation on the lesioned side that leads to the expression of rolling behavior. This persistent descending imbalance during juvenile-to-adult maturation has no long-term influence on spinal network organization and animals never recover an effective locomotor capability. **C:** An acute UL in pre-metamorphic tadpoles also produces an asymmetric descending influence that now persists through metamorphosis (see Lambert et al. 2013). In such an unbalanced developmental environment, however, the adult spinal motor networks are built differently from normal through the establishment of a local asymmetry in propriospinal interactions that are somehow able to counterbalance the asymmetry in the descending commands to allow the restoration of swimming in the post-metamorphic frog. Red arrows: post-UL development; Black arrows: normal development; Double arrow: metamorphic development; Simple arrow: post-metamorphic maturation; Red cross: acute UL; Red dot: persistent UL. The widths of vertical arrows, arrowheads and circuit symbols are proportional to levels of activity.

The results reported here provide evidence that a unilateral removal of labyrinthine sensory inputs in larval *Xenopus* induces adaptive developmental processes in the spinal pathways responsible for dynamic propulsion/posture coupling, the result of which is the expression of a largely restored swimming behavior in the post-metamorphic young adult. Specifically, by taking advantage of the profound remodeling of spinal locomotor circuitry that occurs during metamorphosis ([11]; Figure 8A), we have found that following UL in late pre-metamorphosis, the distributed spinal networks controlling adult hindlimb propulsive movements and body orientation have become asymmetrically coordinated, suggesting the involvement of compensatory processes in the construction of the underlying connectivity during metamorphosis (Figure 8C). In contrast, the same vestibular lesion made in juvenile frogs after metamorphosis had terminated did not lead to an adaptive network response during subsequent maturation to adulthood, and consequently, swimming in these animals remained permanently impaired (Figure 8B).

The processing of labyrinthine, visual and proprioceptive information in the vestibular nuclei plays an important role in the control of both static posture and locomotion [3], [26], [27]. The unilateral loss of vestibular signaling, regardless of age, results in a left-right imbalance in the descending commands to spinal circuitry, in turn leading to specific postural and locomotor impairments that have been characterized in a number of vertebrates [7], [24], [28], [29], including humans [6]. In *Xenopus* also, an acute UL dramatically affects the resting posture and swimming performance of both tadpole and juvenile animals (Figure 1C–D; see also [23]). However, in contrast to terrestrial species [7], [30], [31], including the frog *Rana temporaria* [32], we find that a UL performed in juvenile *Xenopus* is never followed by behavioral recovery, but rather posture and swimming in these lesioned animals remains permanently impaired. In terrestrial animals, recovery of behavioral performance has been attributed to so-called “vestibular compensation” [7], [14] which relies, at least in part, on adaptive plasticity occurring both within brainstem vestibular nuclei [31], [33] and segmental sensorimotor pathways for proprioceptive signaling in the cervical spinal cord [32]. Although we observed modifications in motor output cycle period and burst duration during fictive locomotor episodes in UL66 animals (Table 4), indicating that a unilateral labyrinthectomy may also lead to post-lesional changes in the spinal motor function of post-metamorphic *Xenopus*, other parameters such as resting posture, hindlimb kinematics and muscle coordination, both within and between hindlimbs and between the hindlimbs and dorsal muscles, remained unaltered (Table 2, Figures 2C–D, 3C, 6C and 7C). This in turn suggested that the changes in adult *Xenopus* following a post-metamorphosis UL did not arise from classical vestibular compensation (e.g., [7]) or that, if the latter had occurred, it was insufficient to enable a functional recovery of posture and locomotion (see also [21]).

In contrast, the UL-induced locomotor impairment in pre-metamorphic tadpoles was substantially reduced by the completion of metamorphosis, to the extent that despite a persistent asymmetric resting body posture (Table 2), the swimming performance of these animals was similar to that of intact juvenile frogs (Figure 1C). Neither a kinematic analysis of hindlimb propulsion nor an *in vitro* study of the underlying lumbar motor program provided an obvious explanation for this functional restoration, although dorsal trunk and hindlimb muscle (Figure 6B) and nerve (Figure 7B) recordings did indicate a bilateral asymmetry in trunk-hindlimb coordination. Specifically, in contrast to UL66 frogs, UL54 juveniles displayed an ipsilesional loss of the ascending spinal drive normally responsible for the in-phase coupling between propulsive hindlimb extensions and the contractions of dorsal trunk musculature that are engaged in dynamic postural control during swimming. Significantly, moreover, simulations using a simplified biomechanical model of a UL juvenile indicated that despite its twisted body shape, the recovery of effective locomotion could have indeed resulted from the establishment of such a left-right asymmetry in the animal’s dynamic postural control system (Figure 5). While a contribution of supra-spinal plasticity to the recovery process cannot be fully excluded, it is noteworthy that the posturo-locomotor asymmetry observed in our *in vitro* experiments is expressed under conditions where cerebrospinal pathways are themselves incapable of rhythmogenic bursting (*e.g.*, [34]–[36]; but see [37]), and therefore are unlikely to be responsible for sustaining the functional asymmetry.

It is also unlikely that a UL at larval stage 54 led to a compensatory remodeling of the vestibular system itself since the labyrinthine organs [12] and vestibulospinal projections in frogs [13], [14], [30], [38] are already in place at this stage of the animal’s development. In addition, a UL performed as early as stage 38 in *Xenopus* has been previously found to have no effect on the remaining pre-metamorphic development of the vestibular nuclei [39]. However, recent studies in the adult cat [40], [41] have reported that some neurogenesis, associated with behavioral recovery, can occur in vestibular nuclei in response to a unilateral vestibular neurectomy. Since neurogenesis is widespread in the larval anuran CNS and only ceases after metamorphosis [42], it is conceivable that such a latent developmental process occurring in the brainstem vestibular nuclei during metamorphosis somehow contributes to the locomotor recovery in post-metamorphic UL54 juveniles. Here again, however, the close correspondence of the motor output patterns expressed by the isolated brainstem/spinal cord preparation *in vitro* with our *in vivo* EMG recordings in UL54 animals further suggested that the modified propulsion/posture coupling was not a consequence of compensatory alterations in descending commands or sensory feedback. Rather, the coordination changes were more likely to have resulted from a specific ipsilesional alteration in the ascending lumbo-thoracic coupling pathways that are newly formed during metamorphosis to ensure postural trunk-hindlimb coordination [11]. In contrast to terrestrial quadrupeds, due to the absence of recalibrating limb proprioceptive feedback arising from substrate contact, a UL to larval stages of aquatic *Xenopus* produces a permanent left-right imbalance in vestibulo-spinal influences whereby spinal circuitry on the contralesional side of the cord receives considerably less descending activation than the ipsilesional side [21]. Thus, a diminished influence of ascending lumbar CPG excitation to the ipsilesional thoracic segments could constitute a counter-balancing reaction to the relative increase in brainstem descending drive to the same side of the spinal cord during swimming. Otherwise, the propulsive performance of UL54 juvenile frogs was similar to that of unlesioned animals, both in terms of hindlimb kinematics (Figure 2B–D) and flexor and extensor motor patterns *in vitro* (Figure 3). Altogether, therefore, our findings support the conclusion that, at least in terms of *Xenopus* locomotion, local spinal circuit plasticity underlies the primary adaptation to vestibular sensory deprivation during metamorphosis.

Although sensory information is critical for the formation of sensory systems [43] and is fundamental to the rapid adjustment of ongoing motor programs [3], [27], [44], relatively little is known about the contribution of sensory signaling to the correct assembly of motor circuitry during development [45], [46]. Previous studies in vertebrates [47] (see also for additional pioneering references) and invertebrates [48] have reported near-normal motor behavior in animals that had developed in a restricted sensory environment, suggesting that afferent information plays a relatively minor role in motor network ontogeny. Here also, a unilateral sensory deprivation before and during metamorphosis did not prevent the expression of near-normal swimming in post-metamorphic *Xenopus*. However, our data also strongly suggest that “normality” in the motor behavior of sensory-deficient animals arises only because their propriospinal circuitry had developed differently from that of metamorphosing intact animals. Whereas in the latter, the spinal motor networks develop symmetrically under the influence of bilaterally-equivalent vestibular inputs (Figure 8A; and see [11]), an imbalance in this afferent signaling during the metamorphic period apparently leads to counteractive changes in the assembly of downstream thoraco-lumbar motor circuitry (Figure 8C). On this basis, therefore, our results not only highlight the latent plasticity that can occur in motor network organization during *Xenopus* metamorphosis, but they also point to a fundamental contribution of extrinsically-derived afferent information in shaping this circuit plasticity. This in turn raises the novel and exciting prospect that during development, the appropriate construction of a motor network depends on a precise interplay between an intrinsic ‘representation’ of its target function and the nature of incoming feedback signals from the external environment.
